# An amplified promoter system for targeted expression of calcium indicator proteins in the cerebellar cortex

**DOI:** 10.3389/fncir.2012.00049

**Published:** 2012-07-31

**Authors:** Bernd Kuhn, Ilker Ozden, Yulia Lampi, Mazahir T. Hasan, Samuel S.-H. Wang

**Affiliations:** ^1^Princeton Neuroscience Institute, Princeton UniversityPrinceton, NJ, USA; ^2^Department of Molecular Biology, Princeton UniversityPrinceton, NJ, USA; ^3^Okinawa Institute of Science and Technology Graduate UniversityOnna-son, Okinawa, Japan; ^4^School of Engineering, Brown UniversityProvidence, RI, USA; ^5^Max Planck Institute for Medical ResearchHeidelberg, Germany; ^6^NeuroCure Cluster of Excellence, Charité - UniversitätsmedizinBerlin, Germany

**Keywords:** AAV, cerebellum, crus, FCIP, GECI, *in vivo*, TET, two-photon

## Abstract

Recording of identified neuronal network activity using genetically encoded calcium indicators (GECIs) requires labeling that is cell type-specific and bright enough for the detection of functional signals. However, specificity and strong expression are often not achievable using the same promoter. Here we present a combinatorial approach for targeted expression and single-cell-level quantification in which a weak promoter is used to drive trans-amplification under a strong general promoter. We demonstrated this approach using recombinant adeno-associated viruses (rAAVs) to deliver the sequence of the GECI D3cpv in the mouse cerebellar cortex. Direct expression under the human synapsin promoter (hSYN) led to high levels of expression (50–100 μM) in five interneuron types of the cerebellar cortex but not in Purkinje cells (PCs) (≤10 μM), yielding sufficient contrast to allow functional signals to be recorded from somata and processes in awake animals using two-photon microscopy. When the hSYN promoter was used to drive expression of the tetracycline transactivator (tTA), a second rAAV containing the bidirectional TET promoter (P_tet_bi) could drive strong D3cpv expression in PCs (10–300 μM), enough to allow reliable complex spike detection in the dendritic arbor. An amplified approach should be of use in monitoring neural processing in selected cell types and boosting expression of optogenetic probes. Additionally, we overcome cell toxicity associated with rAAV injection and/or local GECI overexpression by combining the virus injection with systemic pre-injection of hyperosmotic D-mannitol, and by this double the time window for functional imaging.

## Introduction

Genetically programmed protein expression of functional indicators of neural activity (Looger and Griesbeck, 2011) offers the promise of monitoring neuronal networks in action using advanced optical methods (O'Connor et al., 2009). Genetically encoded calcium indicators (GECIs) are sensitive enough to detect small numbers of action potentials (Hendel et al., 2008), including single action potentials under certain circumstances (Wallace et al., 2008; Tian et al., 2009). However, one remaining challenge is achieving expression that is both strong and restricted to particular cell types. Many specific promoters do not drive protein expression strongly enough to allow functional imaging, while the strongest promoters drive expression in many cell types at once. This challenge is made greater by the fact that single-chromophore GECIs are often far below maximum fluorescence at resting calcium levels.

Our model system for pursuing cell type-specific GECI expression is the cerebellar cortex. The cerebellum is a convenient test bed because of its well-organized structure and characteristic cell morphology (Palay and Chan-Palay, 1974) and its history of well-characterized *in vivo* calcium signals achieved largely using bolus loading of synthetic calcium indicators (Sullivan et al., 2005; Hoogland et al., 2009; Nimmerjahn et al., 2009; Ozden et al., 2009; Schultz et al., 2009). Signals can be extracted from Purkinje cell (PC) dendritic arbors, which are non-overlapping, by the covariation of pixels arising from complex spikes (Sullivan et al., 2005; Ozden et al., 2009; Schultz et al., 2009), from interneurons by somatic brightness in the molecular layer (Sullivan et al., 2005; Franconville et al., 2011), and from Bergmann glia by the presence of transglial waves (Hoogland et al., 2009; Nimmerjahn et al., 2009). However, despite this baseline of information, bulk-loading has limits: it does not give good differentiation of neighboring structures or of subcellular domains, and can require advanced image processing (Ozden et al., 2008; Mukamel et al., 2009) to extract meaningful signals. Recently some such problems have been overcome using a cell type-specific adenovirus to drive expression of G-CaMP2 in Bergmann glia (Hoogland et al., 2009) or in utero injection of recombinant adenovirus to label PCs (Yamada et al., 2011).

A common strategy for expressing transgenes in neurons is the use of recombinant adeno-associated virus (Tenenbaum et al., 2004; Shevtsova et al., 2005; Wallace et al., 2008). Using this platform we designed an approach for achieving differential expression that separates a promoter's specificity from its strength. First, we used a recombinant adeno-associated viruses (rAAV) with a strong neuronal promoter that drove strong expression of the GECI D3cpv (Palmer et al., 2006) in all cerebellar cortex interneurons but only weakly in PCs. Second, we used the same promoter to drive a trans-amplification step to achieve boosted expression in PCs but only sparse and weak expression in interneurons. In both cases we determined the absolute expression level of D3cpv and identified a concentration range for neuron viability and *in vivo* signal detection.

A particular advantage of rAAV is its improved diffusion compared with other viruses of larger size. To enhance this advantage, hyperosmotic solution in the form of mannitol can be systemically injected before virus injection to widen extracellular spaces (Burger et al., 2005). In this way mannitol achieves a wider spatial extent of transduction and reduces high multiplicity of viral entry close to the injection site, an event that can lead to excessive protein overexpression and, in the case of GECIs, excessive buffering of intracellular calcium signals. We quantified these advantages and found that mannitol increased the time window over which functional imaging was possible.

## Results

**Figure 1:**
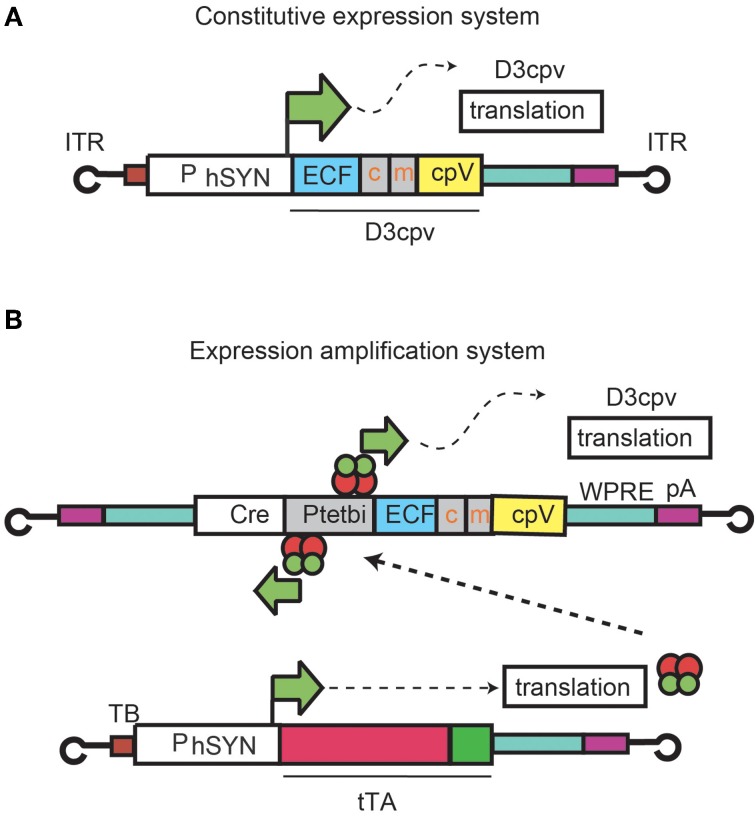
**rAAV expression systems for D3cpv.** Expression under hSYN control **(A)** and an rAAV system consisting of two recombinant viruses where D3cpv is expressed under the control of P_tet_bi activated by the tetracycline transactivator tTA under hSYN **(B)**.

First we describe expression patterns and functional imaging in cerebellar interneurons arising from injection of rAAV2 containing D3cpv under the direct control of human synapsin promoter (hSYN) (rAAV-hSYN-D3cpv) (Figure 1A, Wallace et al., 2008). Next we show expression and functional imaging of PC dendrites using an amplified promoter system in which two rAAVs were co-injected (Figure 1B; Wallace et al., 2008): rAAV-hSYN-tTA to express tTA, a tetracycline-controlled transactivator that is active under absence of a tetracycline analog such as doxycycline; and rAAV-bidirectional TET promoter (P_tet_bi)-D3cpv. rAAV-P_tet_bi-D3cpv contains P_tet_bi, which drives strong expression after tTA binding.

### Expression pattern and functional imaging with rAAV-hSYN-D3cpv

#### Stellate and basket interneurons of the molecular layer

**Figure 2:**
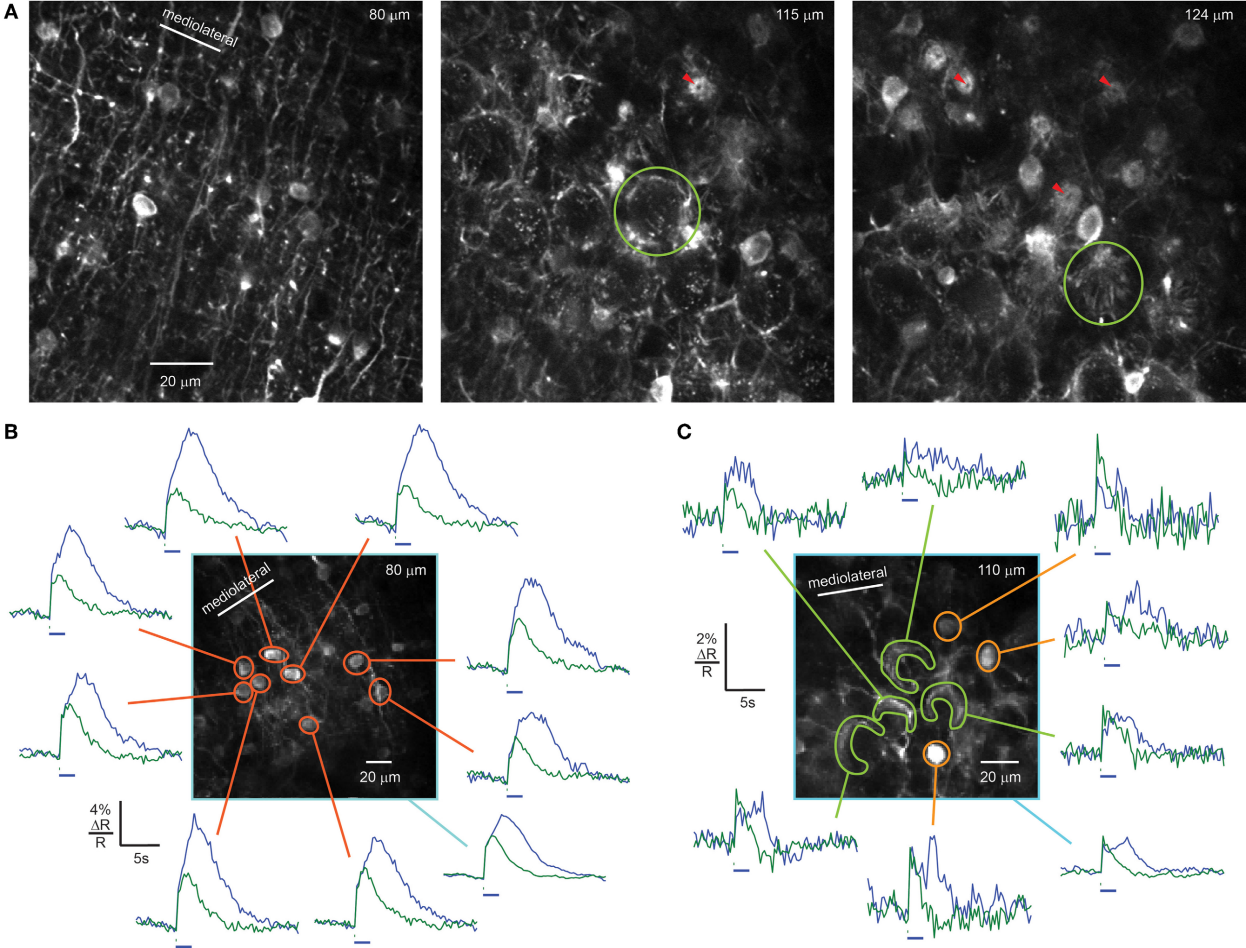
**rAAV-hSYN-D3cpv drives expression in stellate and basket cells of the molecular layer. (A)** Brightly labeled dendrites, somata, and axons of stellate and basket cells. Basket cell axons form dense networks around the somata of PCs (middle, light green circle). Axonal fibers form bundles (right, green circle) converging on the initial segment of the PC axons forming pinceaux. The PC axon is visible as dark dot in the center of the pinceau cross sections (red arrow heads). **(B)** Sensory-evoked calcium signals recorded in crus II of an awake mouse in response to airpuff stimulation (100-ms airpuff green, 2-s airpuff blue, 48 averages) of the snout in the molecular layer. Orange masks indicate stellate cell somata 40 μm above the PC layer. **(C)** In the PC layer, calcium signals from axons of basket cells can be recorded under same conditions (40 averages). Green and yellow masks indicate baskets and basket cells, respectively. The average signal of the field of view (light blue) shows similar response as single cells **(B,C)**. Horizontal bars, 100-ms and 2-s airpuff. Numbers in the upper right corner indicate imaging depth.

Three to four days after injection of rAAV-hSYN-D3cpv into the cerebellar cortex, we found strong, high-contrast fluorescence in molecular layer cell bodies and their processes. Somata (diameter 7.5 ± 1.0 μm, *n* = 30) were surrounded by a dense network of dendrites and axons oriented mainly parasagittally, with shorter processes extending mediolaterally, as expected from morphological studies (Palay and Chan-Palay, 1974). The overall expression pattern resembled a one-dimensional grating (Figure 2A, left). The cell body density decreased with greater distances from the PC layer, consistent with the distribution observed in this cerebellar region using bolus loading (Sullivan et al., 2005). In areas where expression spanned the entire molecular layer, the overall density of somata was 82 ± 6 per 10^6^ μm^3^ (3 animals), comparable to the reported density in mouse of 84 ± 8 stellate/basket interneurons per 10^6^ μm^3^ (Sturrock, 1989) and indicative of a high infection probability at the injection site.

In the PC layer, interneuron processes completely ensheathed PC somata with fluorescent puncta (Figures 2A middle, 3A, comparable to puncta in Hasan et al., 2004). PC ensheathments comprised bundles of basketlike fibers arising from multiple neurons that condensed at the boundary of the Purkinje and granule cell layer to form a conical plexus, called a “pinceau” (Palay and Chan-Palay, 1974). Pinceaux (Figure 2A, green circles) had a diameter at the PC soma of 18.1 ± 2.0 μm and ended 12.5 ± 0.9 μm away from the PC soma (*n* = 8). At the center of each pinceau was a void along the axis 1–2 μm wide coincident with the initial segment of the PC axon (Figure 2A, red arrowheads).

**Figure 3:**
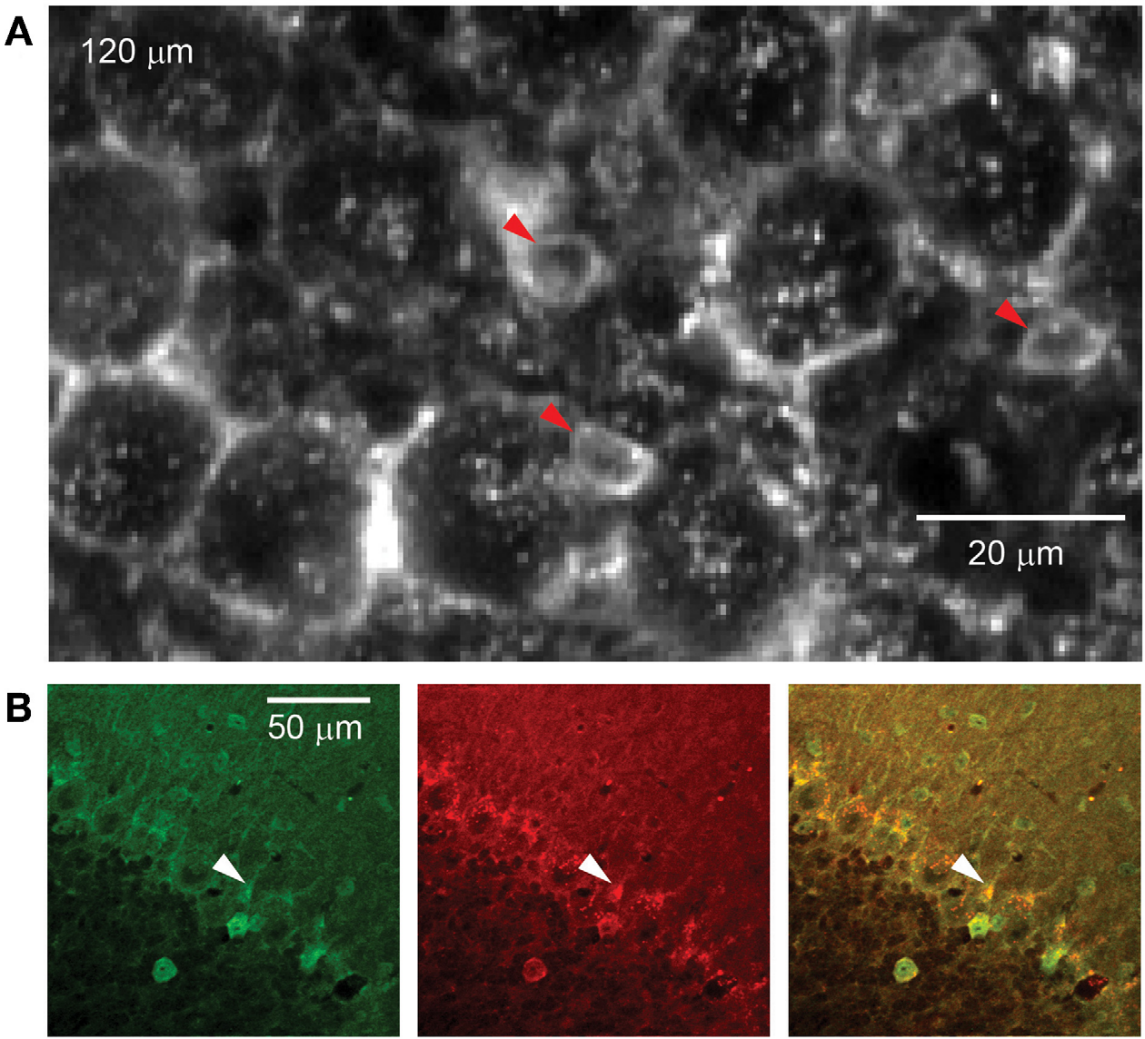
**rAAV-hSYN-D3cpv labels candelabrum cells of the PC layer. (A)** The somata of candelabrum cells indicated by arrowheads are found between unlabeled Purkinje somata and brightly labeled axonal baskets. **(B)** Double labeling (superposition yellow) with anti-GABA (green) and anti-glycine (red) confirms identification as candelabrum cells. Number in the upper left corner indicates imaging depth.

To test the sensitivity of D3cpv to neuronal activity, we recorded changes in the green/blue ratio R (ΔR/R_0_) while stimulating the snout of head-fixed mice with 100-ms or 2-s airpuffs, which are known to trigger complex spike responses in crus II (Brown and Bower, 2001). Functional signals in interneurons were absent in anesthetized mice, as previously reported (Franconville et al., 2011). In awake animals, at airpuff intensities below those that evoked a visible startle, we recorded average stimulus-triggered responses from single somata of stellate cells in the upper three-quarters of the 110-μm-thick molecular layer (Figure 2B, orange masks), interneurons close to the PC layer (Figure 2C, yellow masks), and baskets surrounding PC somata (Figure 2C, light green mask). Brief (100 ms) airpuffs evoked rapid rises in fluorescence in molecular layer somata (Figure 2B) and baskets (Figures 2B,C, green traces). The rise began in all bright structures in a 250 μm by 250 μm field of view within the first 256-ms frame after the 100-ms stimulus onset. In baskets the signal reached the maximum during the first 256-ms frame. In the somata of molecular layer interneurons the signal reached maximum amplitude 1.3 ± 0.2 s (*n* = 9) after stimulus onset, with 74 ± 11% of the increase occurring within the first frame. Taken together, these results indicate that under awake conditions, facial airpuffs broadly activate many crus II stellate/basket interneurons.

Signals from baskets peaked at the end of the airpuff and declined rapidly. The falling phase of signals was slower in somata (*t*_1/2_ = 2.0 ± 0.3 s) than in baskets (1.4 ± 0.7 s). The fall time in baskets was consistent with the intrinsic off-response for D3cpv of τ = 2.0 s, i.e., *t*_1/2_ = 1.4 s (Palmer et al., 2006), suggesting that in small structures such as axon terminals, temporal resolution was limited by indicator dynamics, whereas somatic signals integrate calcium entry over seconds due to a low surface-to-volume ratio (Sala and Hernández-Cruz, 1990). However, peak ΔR/R signals were higher in somata (5.6 ± 0.4%) than in baskets (2.5 ± 0.4%), suggesting that the soma receives calcium from additional sources such as NMDA receptors (Franconville et al., 2011) or calcium release from internal stores. Prolonged response kinetics were apparent for 2 s-long airpuffs, which evoked signals with a continuously rising phase (Figure 2B, blue trace).

#### Candelabrum cells

Between PC somata we found fluorescent, pear-shaped somata with thick apical dendrites that led toward the molecular layer where they then branched (Figure 3A). They were found at a density of 283 per mm^2^, in a ratio of 1:6.6 to PCs. The average soma diameter was 5.8 ± 1.2 μm (long axis 6.7 ± 0.9 μm, short axis 4.8 ± 0.7 μm, *n* = 8). These structures resembled candelabrum cells, a neuron type originally found in rats (Laine and Axelrad, 1994) and confirmed in primates (Crook et al., 2006). We used immunohistochemical double-labeling to demonstrate co-localization of GABA and glycine (Figure 3B), a characteristic of candelabrum neurons (Crook et al., 2006).

#### Lugaro and globular cells

**Figure 4:**
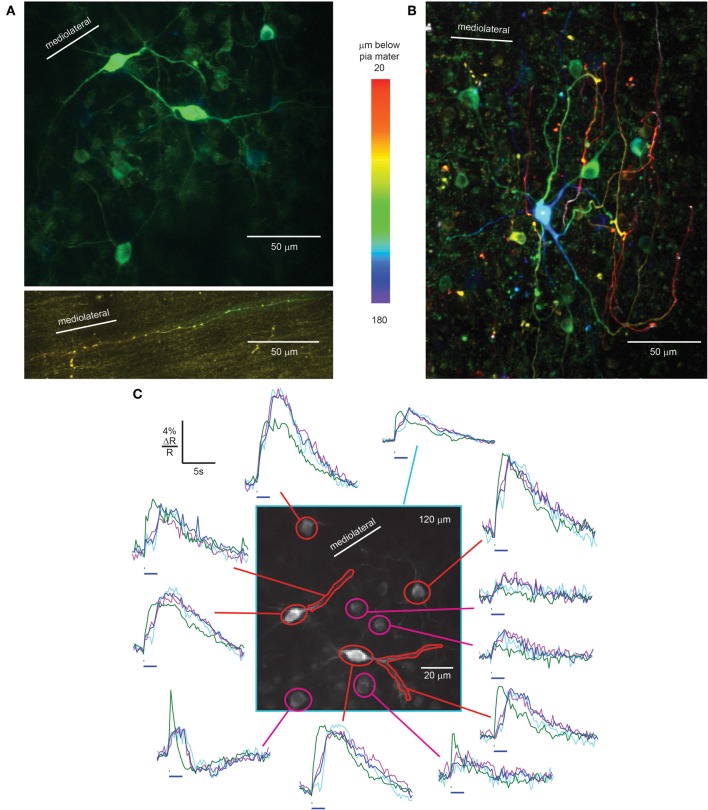
**rAAV-hSYN-D3cpv labels Lugaro and Golgi cells of the granular layer. (A)** Fusiform Lugaro cells in the granular layer, just below the PC layer, with X shaped dendrites in a plane parallel to the PC layer. The Lugaro cell axon courses in the lower part of the molecular layer parallel to the parallel fibers (bottom). **(B)** Golgi cells with dendrites ascending to pia mater. The color code in **A** and **B** indicates depth below pia mater. **(C)** Sensory-evoked calcium signals recorded in the granular layer from Lugaro cell somata and dendrites (red) and Golgi cells (magenta) in response to 100 ms (green trace) and 2-s airpuffs (blue trace), 40 averages. Taking behavior during the 2-s airpuff into account, the purple trace shows the average calcium transient with constant body movement and whisking (20 averages) while the violet trace shows the calcium transient where body movement stopped during the airpuff (17 averages). Horizontal bars, 100-ms, and 2-s airpuff. Number in the upper right corner indicates imaging depth.

In the granule cell layer just below the PC layer, we identified Lugaro cells by their fusiform somata, X-shaped dendritic trees in a plane parallel to the PC layer (Figure 4A, center, Laine and Axelrad, 1996), and brightly labeled axons in the molecular layer (Figure 4A, bottom) extending along the parallel fiber axis (Laine and Axelrad, 1996). Granule cells were poorly expressing and therefore excluded as the origin of these axons. Also seen at this level were rounded cells with relatively small somata that did not meet the criteria for Lugaro cells, but could have been globular cells (Laine and Axelrad, 2002; Simat et al., 2007) or small Golgi cells (Simat et al., 2007).

In awake mice, facial airpuff stimulation evoked fluorescence increases in Lugaro cell somata and dendrites. As was the case for molecular layer interneurons, signals were absent in anesthetized mice. A 100-ms airpuff stimulus caused a sharp increase during the stimulus, a plateau lasting several seconds, and a slow decrease (Figure 4C, green traces of red masks). Calcium signals were up to 7.2% ΔR/R in response to 100-ms airpuffs. Responses to 2-s airpuffs revealed fast-rising signals at the start of the puff, a continuing slower increase for the first 1.3 ± 0.2 s of the puff. Then, surprisingly, the signal increased steeply, continued to increase after the puff offset until it reached a plateau and eventually slowly decreased (Figure 4C, blue trace of red mask). In Lugaro cell dendrites the plateau was absent. To explain the sudden signal increase during the stimulus we analyzed the mouse behavior recorded during the imaging session of Figure 4C (average of 40 trials). The movies showed body movement and whisking during the 2-s puffs in 25 out of 40 trials while in 17 out of 40 trials the body movement stopped during the airpuff. The body movement stopped on average 0.9 ± 0.4 s after stimulus onset. This behavioral difference is reflected in the calcium traces of Lugaro cells (purple and light blue, respectively). While constant body movement during the puff was associated with a monotonic calcium increase in Lugaro cells, the stopping of movement correlated with a dip in calcium before rising again. For further investigation of the correlation between neuronal Ca^2+^ signals in interneurons and behavior it will be crucial to achieve better signal-to-noise Ca^2+^ recordings with improved GECIs to overcome averaging. Additionally, calcium imaging should be paired with electrophysiological recordings to reveal the underlying electrical activity.

#### Golgi cells

The second major interneuron type of the granular layer is the Golgi cell (Palay and Chan-Palay, 1974). Scattered throughout the granule cell layer we found strongly expressing large Golgi cells, identified by their large somatic diameter, 12.4 ± 1.2 μm (*n* = 13). Consistent with past characterization, Golgi cells dendrites could be followed up to the molecular layer and in some cases almost up to the pia mater (Figure 4B), features that distinguished them from Lugaro cells. Cells with diameter intermediate between large Golgi cells and granule cells also expressed D3cpv, consistent with either small Golgi cells or globular cells (Laine and Axelrad, 2002).

As expected from the diversity of Golgi cell subtypes (Simat et al., 2007), putative Golgi cells showed stimulus-evoked signals with a range of amplitudes and kinetics. 100-ms airpuffs caused calcium responses (Figure 4C, green trace of magenta masks) with ΔR/R of up to 7.8 ± 0.4%. 2-s airpuff stimulation resulted in steep signal onsets, a slow rise during the puff and an immediate decrease time-locked to the puff offset (Figure 4C, blue trace of magenta masks). In some cases sharp increases occurred at both stimulus onset and offset (Figure 4C, right trace at bottom). The behavior-based averages (constant body movement: purple trace, movement stops: light blue trace) were similar within the constraints of the limited signal-to-noise ratio. Again, improved GECIs and combined optical and electrical recordings will open the door for future investigations.

#### Purkinje cells

We did not find PCs expressing D3cpv at comparable levels to interneurons in any of the crus II injected mice (*n* = 25). No fluorescence labeled PC dendrites could be detected. Fluorescence intensity in PC somatic cytoplasm including puncta was lower than in basket cell ensheathment of PCs by a factor of 8 ± 4 (*n* = 14) during the first four weeks after infection. Thus in PCs, hSYN is a weak but not silent promoter.

### Expression in purkinje cells with rAAV-hSYN-tTA and rAAV-P_tet_bi-D3cpv

**Figure 5:**
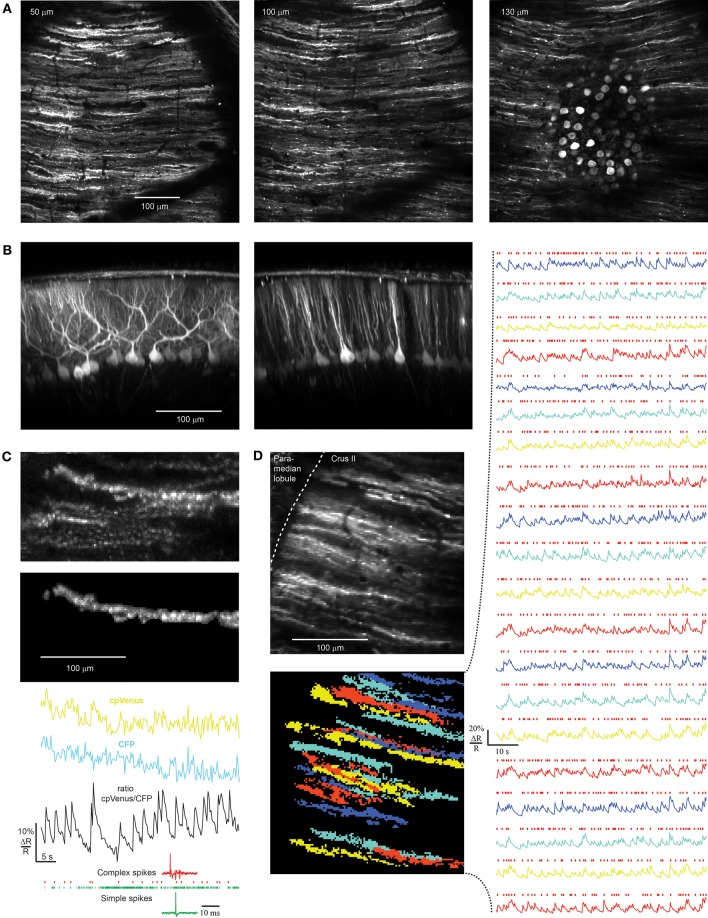
**rAAV-hSYN-tTA and rAAV-P_tet_bi-D3cpv labeling of PCs and recording in awake mice. (A)** Near-ubiquitous labeling of PCs within a field of view. **(B)** Reconstruction of the parasagittal orientation of PCs. **(C)** Transients representing PC complex spikes as shown by simultaneous electrical (bottom) and ratiometric optical recording (black trace). In the corresponding raw traces of CFP and cpVenus the functional signals are hidden in movement artifacts (mask of PC dendrite: top). The dendrite bleaches in both channels 12% during the 50 s of functional imaging. Only complex spikes (red marks of electrical recording) but not simple spikes (green marks) correlate with functional calcium signals. **(D)** Population activity of PCs were measured with D3cpv at the edge of the folium so that dendrites are seen from the side during quiet wakefulness. Because of different expression levels the signal-to-noise ratio varies across the PC population from 3 to 10. Masks were selected by an automated algorithm applying spatial independent component analysis. Red marks indicate complex spike events. Numbers in the upper left corner indicate imaging depth.

Co-injection of rAAV-hSYN-tTA and rAAV-P_tet_bi-D3cpv resulted in brightly labeled PCs (Figures 5A,B), identifiable by their neatly aligned, parasagittally oriented dendrites which had no overlap with one another (Palay and Chan-Palay, 1974; Ozden et al., 2008, 2009; Mukamel et al., 2009; Schultz et al., 2009). In a 250 μm by 250 μm field centered around the injection site a high percentage of PCs were labeled, as could be judged from the soma-filled PC monolayer and dendrite-filled molecular layer. The cell density of 17.9 ± 1.2 cells per 10^4^ μm^2^ and soma diameter of 16.7 ± 2.1 μm (*n* = 14) confirmed previous results in mouse (Sturrock, 1989). Brightly labeled axons descended into the granular layer. Somatic intensity levels varied over a 10 ± 2-fold range from the dimmest to the brightest cell (*n* = 5 animals).

In contrast, fluorescent cells in the molecular and granular layer were sparse and dim. The fluorescent intensity of the brightest stellate/basket cell somata was 5 ± 3 times lower than that of nearby PC dendrites (intensity of 8 molecular layer somata in three injections compared with 24 PC dendrites). Also, the brightest stellate/basket cells expressing under the tTA/TET promoter system were about three times dimmer than typical stellate/basket cells under the hSYN promoter.

Injection of rAAV-P_tet_bi-D3cpv or rAAV-hSYN-tTA alone led to no detectable expression in any cells (*n* = 3 injections each), demonstrating that expression of tTA under the otherwise weak hSYN is necessary for neuronal labeling and not from leakiness of P_tet_bi acting alone.

Simultaneous extracellular electrical and optical recording demonstrated that calcium transients in PC dendrites had the frequency and time course expected from previous measurements of complex spikes *in vivo* (Figure 5C, bottom). In brightly labeled neurons we found ΔR/R of 10% for single complex spikes with a rise time less than 256 ms and fall time *t*_1/2_ of 1.79 ± 0.36 s, reflecting the slow off-response of D3cpv (Palmer et al., 2006; Hendel et al., 2008). Optical functional signals recorded in awake animals were usually masked by movement artifacts and could be observed only in ratiometric traces (Figure 5C, top). Complex spikes occurred at a rate of 0.61 ± 0.12 Hz (*n* = 68 dendrites, 6 mice) under awake but quiet conditions as previously reported (0.57 ± 0.39 Hz; Flusberg et al., 2008). The signal-to-noise ratio was 5.5 ± 2.6 ranging from 3 to 10 (*n* = 68 dendrites, six mice). In awake optical recordings paired with electrical recordings during combined movement and non-movement periods, 94.3 ± 1.3% of electrically recorded complex spikes were detected (*n* = 238 complex spikes in 4 dendrites). This allowed us to record spontaneous population activity of PCs with high reliability from dendrites in awake mice (Figure 5D). In the example shown (Figure 5D) PC dendrites were imaged at the edge of the folium so that dendrites were seen from the side. Judging from the 3D reconstruction of the area we recorded 20 of the estimated 40 dendrites in the field of view. In general, the percentage of recordable dendrites depended strongly on depth of rAAV injection, speed of rAAV injection, and virus quality and concentration. An infection rate of almost 100% could be reached (Figure 5A) but because of the variability of expression levels functional imaging was achieved in only ~50% of the PCs in a 250 μm × 250 μm field of view, somewhat lower than with in utero electroporation (50–80%, Yamada et al., 2011) but considerably higher than chronic expression of GCaMP3 using Ai38 mice (Zariwala et al., 2012).

### Probe expression and time course extension with D-mannitol

To monitor the time course of D3cpv expression, we mounted a chronic cranial window at the time of virus injection. Imaging began 3 or 4 days after injection and every 2–3 days thereafter. To quantify the apparent intracellular D3cpv concentration, we lowered a pipette standard filled with a known eGFP concentration into the brain at the location of D3cpv expression. Care was taken to adjust viewing conditions so that the focal plane passed through the center of the soma and a comparably-sized segment of pipette, in both cases fully spanning the excitation volume of the two-photon point spread function. Since the fluorescence intensity of cpVenus is independent of the calcium concentration if excited directly by 940 nm light and not through Förster resonance energy transfer from CFP, comparison with cell brightness allowed estimation of the protein concentration. This estimate of probe concentration is approximate because probe qualities such as chromophore environment and posttranslational modification may differ between neuronally-expressed D3cpv and pipette-loaded eGFP.

For rAAV-hSYN-D3cpv injection, we found brightly labeled cells with increasing expression up to 6 ± 1 days after injection (*n* = 4 mice). In basket, stellate, Lugaro, and Golgi cells with normal morphology (Palay and Chan-Palay, 1974) we found typical apparent D3cpv concentrations of 50–100 μM, with individual cells expressing up 300 μM. On later days we found axonal blebbing which was especially apparent in baskets, followed by cell death within 2–4 days. This blebbing was observed both in repeatedly imaged locations, where photodamage from previous sessions may have occurred, and in locations that had not been previously imaged. The blebs of basket cell axons and somata of swollen Lugaro cells had apparent D3cpv concentrations of up to 600–800 μM, respectively.

**Figure 6:**
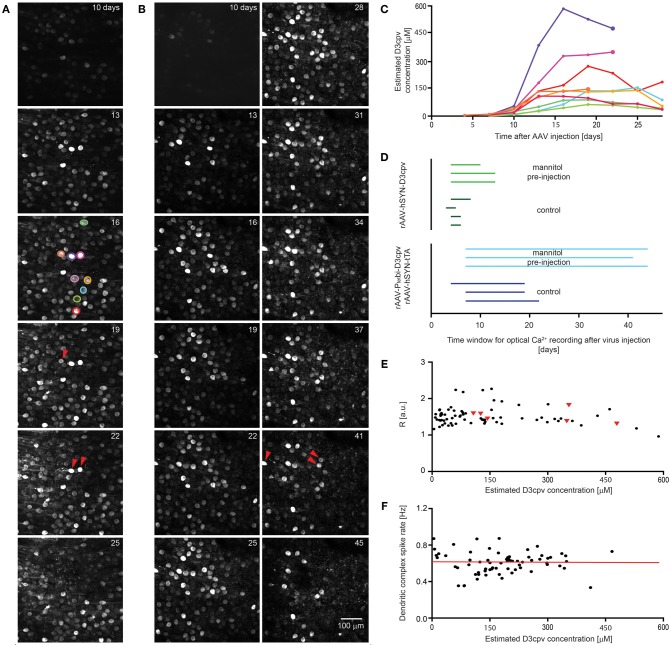
**Extension of time window for imaging by systemic mannitol pre-injection.** Images showing the time course of expression of D3cpv **(A)** without and **(B)** with systemic mannitol injection. Red arrowheads indicate cells that disappear in the next image taken 3 days later. **(C)** Time course of estimated D3cpv concentrations of single neurons indicated in **(A)** by color show no clear correlation between D3cpv concentration and cell death within the following 3 days indicated by a larger symbol. **(D)** The time window for calcium imaging in PCs and interneurons in the cerebellum doubles with systemic mannitol injection. Each line, representing one animal, starts when expression was high enough for functional imaging and ends 2–3 days before the first morphological change in neuronal processes. In the case of interneurons imaging was possible at first observation, 3–4 days of recovery after virus injection. **(E)** Intensity ratios measured at the soma are a measure for somatic calcium levels and are plotted over the estimated D3cpv concentration in the PC somata. PCs which disappear during the following 3 days were found at a wide range of intensities (red triangles). Intensity ratios were taken from time courses shown in **(A)** and **(B)**. **(F)** Dendritic complex spike rate was independent of expression level in the estimated concentration range analyzed. Red line indicates linear regression.

For the rAAV-hSYN-tTA and rAAV-P_tet_bi-D3cpv system we found a similar, but slower time course. The first PCs were visible after 4 days and expression increased steadily up to 20 ± 2 days and then decreased slowly (Figures 6A,C). A few labeled PCs then started to disappear (Figures 6A,C). PCs typically used for functional imaging of complex spikes had apparent D3cpv concentrations of 10–300 μM (Figure 6F), while the brightest cells appearing at later time points had apparent concentrations of ~600 μM (Figure 6E).

A potential cause of local cell death was retention and concentration of viruses close to the injection site. To test this we injected hyperosmotic D-mannitol solution systemically 15 min before virus injection. Cell toxicity was delayed, with blebbing of interneuron axons occurring later and PCs surviving longer (Figure 6B). The time window for imaging increased from 6 ± 1 to 12 ± 2 days for rAAV-hSYN-D3cpv injection and from 20 ± 2 to 43 ± 2 days for combined rAAV-hSYN-tTA and rAAV-P_tet_bi-D3cpv injection (Figure 6D). In PCs the expression level peaked with and without D-mannitol at 22 ± 1 and 26 ± 3 days after injection (*n* = 3 animals for each condition).

We assessed whether perturbations of calcium concentration or signaling could predict PC toxicity. PC toxicity (Figure 6D, red triangles) was scored as occurring if a PC that was initially visible could not be found 3 days later. Bright PCs sometimes survived, while cells with expression of less than 150 μM sometimes disappeared. Resting fluorescence ratios *R*, which reflect Ca^2+^ concentration (Figure 6E, *n* = 74 cells of time courses shown in Figures 6A and B) did not predict cell survival. Finally, the complex spike rate in awake animals (*n* = 68 dendrites of 6 mice), which reflects both climbing fiber innervation and PC dendritic function, did not depend on D3cpv expression level (slope of linear regression: 0.02 ± 0.15 Hz/M, Figure 6F). In summary, neither D3cpv expression level nor parameters of calcium signaling were predictive of PC disappearance, suggesting that cell death occurs by some mechanism independent of the indicator protein itself.

## Discussion

### Expression pattern under hSYN and hSYN/ P_tet_bi control delivered by rAAV2/1

hSYN is a general neuronal promoter and it has previously been shown that the rAAV2/1-hSYN system can be used to achieve expression in both excitatory and inhibitory neurons in neocortex (Nathanson et al., 2009). In cerebellar cortex, however, we find that hSYN is highly heterogeneous with respect to cell type. Expression was an order of magnitude stronger in interneurons than in PC somata while PC dendrites were not observable. This difference was sufficient to allow high-contrast imaging of interneurons *in vivo*. The combination of a weak promoter, in our case hSYN in PCs or alternately a true cell-type specific promoter, and a second, amplifying promoter provides the converse specificity. In our case the expression level in PC somata was increased by more than 10-fold compared with either hSYN or the amplification promoter system alone. This amplification resembles patterns of expression found in cerebral cortex and hippocampus (Wallace et al., 2008). One important prerequisite must be fulfilled: the second, amplifying promoter must be permissive in the target cell, in this case P_tet_bi in PCs. Our results show that evidently, P_tet_bi is barely permissive in cerebellar cortex interneurons.

### Cell-type targeting in the cerebellum for functional imaging

In cerebellar cortex our previously published approaches allowed labeling and recording from Bergmann glia and velate protoplasmic astrocytes with recombinant adenovirus (Hoogland et al., 2009). The current work adds stellate, basket, candelabrum, Lugaro, and Golgi cells with rAAV-hSYN-D3cpv, and PCs with the rAAV-hSYN-tTA and rAAV-P_tet_bi-D3cpv system. In the present study, for functional imaging we identified a useful apparent concentration of 10–300 μM, setting a target for future approaches and other promoters. Tools for targeted cell-type-specific expression are especially useful for sparse cell types like Lugaro, candelabrum, and globular cells which are difficult to identify by less-specific means of dye loading or GECI expression. In future interneuron experiments it will also be important to combine electrophysiological and calcium recordings for a better understanding of the network activity. The PC labeling pattern we observe should be useful for applications requiring repeated imaging over many days (Huber et al., 2012) or high signal-to-noise to allow detection of subdendritic events. A higher homogeneity of expression in PCs might be achievable with injections larger than the small ones (35 nL) used here. Reliable population activity within a single imaging session should be recorded with synthetic dyes, which label 100% of cells with lower contrast (Sullivan et al., 2005; Flusberg et al., 2008; Ozden et al., 2009; Schultz et al., 2009).

### Endogenous buffers and GECI kinetic limits

In this study we used the GECI D3cpv, which was effective for imaging PC complex spikes. For awake imaging, the fact that D3cpv comprises two fluorophores allowed the fluorescence ratio to be used for monitoring calcium, thus overcoming movement artifacts (Figure 5C). GECIs with higher signal amplitude and faster kinetics (Looger and Griesbeck, 2011; Tian et al., 2012) could be used too if they were co-expressed with a different-color fluorescent protein such as mCherry to allow red/green ratioing.

A challenge to using calcium as a readout of neural activity arises from the presence of endogenous calcium binding. All cytoplasm contains baseline low-affinity calcium buffering (of unclear molecular identity) that gives a minimum bound: free ratio (κ) in the range of 30–100, attenuating the amplitude and slowing the time course of calcium signals. In addition to this, exogenously introduced GECIs and endogenous calcium binding proteins add further buffering. In PCs, of particular interest is the fast-calcium-binding protein calbindin D28k, which binds 4 calcium ions per molecule with high affinity (*K*_*D*_ = 0.4 μM). Calbindin-D28k is expressed in PCs at concentrations of 100–360 μM (Schmidt, 2012) and attenuates peak calcium concentration arising from a single complex spike (Schmidt et al., 2003). The additional buffering (Δκ) contributed by calbindin is expected to heavily influence PC calcium signaling. At a resting calcium concentration of 0.1 μM calbindin's contribution to bound: free ratio would be expected to be Δκ ~ 4 [calbindin]/*K*_*D*_ = 1000–3600, far greater than the contribution of D3cpv of Δκ = 400–800 (assuming *K*_*D*_ = 0.5 μM and [D3cpv] = 50–100 μM with 4 sites per molecule). Thus, the additional perturbation of calcium dynamics from D3cpv is likely to be relatively minor. Consistent with this, in our experiments the somatic calcium concentration in PCs was not strongly dependent on apparent D3cpv concentration (Figure 6E).

In molecular layer interneurons, the slow calcium-binding protein parvalbumin may have contributed to the need to average multiple trials to obtain usable signals. Parvalbumin is present in Purkinje (Schmidt et al., 2003) and basket (Kosaka et al., 1993; Collin et al., 2005) neurons at concentrations of ~100 μM in soma and dendrites and ~1 mM in axons. Although the amount by which parvalbumin attenuates calcium changes is potentially relatively small because much of it is bound to magnesium, its effect would be largest in axons and baskets. Even in PC dendrites, where its concentration is lower, its removal in knockout animals affects the time course of calcium clearance in the tens of milliseconds following a spike (Schmidt et al., 2003), indicating that it competes for entering calcium ions on that time scale. In this way parvalbumin could impede spike rate readout by temporal filtering of activity-dependent changes in calcium concentration.

Finally, D3cpv itself, like other GECIs, imposes intrinsic limits on the ability to report spike rates and times. All GECIs currently in use have positive cooperativity, so that the reportable calcium concentration range is narrow for any one GECI, and calcium-binding saturation occurs easily. In addition, D3cpv has slow response dynamics: off-responses have a time constant of approximately 2 s (Figure 5C; Hendel et al., 2008). Several GECIs have faster off-responses, GCaMP2/3/5 and TN-XL (Hendel et al., 2008; Tian et al., 2009). A further impediment is the slow on-response of GECIs arising from binding or intramolecular dynamics. Improvements in GECI on- and off-response speeds are under development (Sun et al., 2011) and should eventually allow firing rate changes to be monitored more effectively.

### Effect of systemic co-administration of D-mannitol on rAAV infection

Finally, we have demonstrated that systemic D-mannitol both increases the viability of cells near the virus injection site. rAAV capsids have a typical diameter of 18–26 nm, smaller than the intercellular spaces in cerebellum of 38–64 nm (Thorne and Nicholson, 2006). Spread of virus particles is likely to proceed by increasing intercellular space further, which would reduce the local peak of virus concentration close to the pipette tip both during and after injection. The wider distribution but lower concentration of virus particles at the injection site causes a slower onset of expression due to the lower multiplicity of viral entry. In this way systemic mannitol injection doubles the time window for chronic imaging.

## Methods

### Recombinant adeno-associated viruses

All viruses used were previously described (Wallace et al., 2008). D3cpv was cloned into either pAAV-hSYN or pAAV-P_tet_bi-Cre expression plasmids and tTA into pAAV-hSYN. Viruses were generated by co-transfecting different viral constructs with pDp1, pDp2 (ratio: 3:1) helper plasmids in HEK293 cells. Seventy-two hours after transfection, HEK293 cells were collected and viruses purified. Viruses were purified on an iodixanol gradient and infectious virus titers were determined in primary neuron cultures and were 1–5 × 10^8^ transducing units per microliter.

### Viral injection and chronic window implantation

All animal experiments were approved by the Princeton Institutional Animal Care and Use Committee. Virus injection was done as previously described (Kuhn et al., 2011). C57/Black6 mice, age 25–40 days, were anesthetized with ketamine/xylazine (Sigma) and injected with carprofen and dexamethasone (Sigma). A 3-mm diameter craniotomy was opened exposing a part of crus II on the right hemisphere and leaving the dura mater intact. The coordinates were 7.0 mm posterior of bregma, 2.2 mm lateral, and 2.0 mm ventral. 35 nL of viral stock solution was injected 160–180 μm below the pia mater (MP-285, Sutter Instruments) with a beveled, tick-marked quartz pipette (inner diameter: 0.3 mm, meniscus movement of 0.5 mm) over a span of 10–15 min. A 5-mm diameter round glass cover slip was mounted on the craniotomy and sealed with cyanoacrylate glue avoiding any contact with the dura mater. Additionally, an aluminum head post was glued to the skull. Dental acrylic was used to build up a small well around the glass cover slip including the head post for stability and immersion water.

### Viral injection with D-mannitol co-administration

In some experiments D-mannitol (15% in PBS, 3 ml per 100 g body weight) was injected intraperitoneally 15 min before the virus injection. In these cases the chronic cranial window was mounted 24 h after the injections. The animals were allowed to recover from the virus injection and window mounting for 3 or 4 days before the start of imaging.

### *In vivo* two-photon imaging

Microscopy was performed using a custom built two-photon microscope (MOM, Sutter) based on the design of Winfried Denk and running ScanImage (Pologruto et al., 2003). The excitation wavelength was 850 nm of a Ti:sapphire laser (MaiTai, Spectra Physics). A 20×/1.0 N.A. water immersion objective (Zeiss) and two GaAsP photomultiplier tubes (Hamamatsu) were used. Fluorescence was detected simultaneously in the green (520–600 nm bandpass filter, Chroma) and blue (460–500 nm bandpass filter, Chroma) wavelength range of cpVenus and cyan fluorescent protein (CFP), respectively. Laser power for functional imaging of PCs and molecular layer interneurons was up to 50 mW, and granular layer interneurons up to 100 mW. The ratio of cpVenus and CFP fluorescence was used to analyze functional signals. Ratiometric imaging was done to eliminate movement artifacts when recording from awake animals. At the beginning of the imaging session, animals were anesthetized with 1.5% isoflurane in O_2_ and then headfixed under the microscope. Stacks for three-dimensional reconstruction were acquired under anesthesia (1.0–1.5% isoflurane) with 512 by 512 pixels and 4 or 9 averages. Animals for analysis of expression time course were not used for functional imaging to avoid potential damage by bleaching or phototoxicity. For the time courses stacks were acquired every 2 and 3 days for interneurons and PCs, respectively. Functional imaging was done under awake conditions sitting on a platform with isoflurane/O_2_ flow off for more than 5 min. Behavior was recorded with an infrared camera (DCR-DVD308 DVD Handycam, Sony) and infrared illumination (HVL-HIRL Video/IR Combo Light, Sony). Two-photon movies were acquired with 128 by 128 pixels and 2 ms per line (256 ms/frame, a rate of 3.9 Hz) and data were analyzed with MATLAB and ImageJ.

Masks of PC dendrites were found using spatial independent component analysis (sICA) implemented with the FastICA algorithm (Hyvarinen and Oja, 2000). The movies were first converted to ΔR/R movies by calculating and registering time dependent ΔR/R values for each pixel. sICA was run on these ΔR/R movies with 10 (for movies with up to 5 dendrites) or 50 (for movies more than 10 dendrites) spatial components where the contrast function was a Gaussian approximation to negentropy (Hyvarinen and Oja, 2000). This procedure separated individual dendrites to individual sICA components. However, not all the sICA components carried a dendrite. sICA components carrying a dendrite were determined by visual inspection. Usually there was no ambiguity in this procedure since such sICA components consisted of a clear group of high intensity pixels in the shape of a dendrite (Ozden et al., 2008) over a low intensity background. To extract the mask of a dendrite from its sICA component, the sICA component was thresholded at a level (determined manually) so that bright pixels belonging to the dendrite is set to 1 (above threshold) and the rest to 0 (below threshold). The threshold values varied significantly between movies and depended on the signal-to-noise ratio. However, the final dendrite masks obtained with this method had good separation for individual dendrites with little overlap between them.

### Extracellular recordings of PCs

Extracellular single-unit recordings from PCs were made using glass micropipettes pulled to 6–10 MΩ with a P-2000 puller (Sutter Instruments) and filled with artificial cerebrospinal fluid containing (in mM) 135 NaCl, 5.4 KCl, 5 NaHEPES, 1 MgCl_2_, and 2 CaCl_2_ (pH 7.3 with HCl). To introduce the electrode a 200 μm opening was carefully drilled into the glass at the edge of the window. Care was taken not to heat the glass by friction. The window glass was regularly cooled using canned air and the drill bit by immersion into water. The glass micropipette was positioned under anesthesia, then isoflurane terminated. When mice started to whisk and move, typically within 10 min after isoflurane termination, recordings were started. Signals were acquired using a NeuroData IR-283A amplifier (Cygnus Technology), amplified 10–100-fold, band-pass filtered at 0.3–10 kHz with a Brownlee Model 440 amplifier (Brownlee Precision) and saved to a personal computer equipped with a data acquisition board (PCI-MIO-16E-4, National Instruments) and custom MATLAB software.

### Estimation of D3cpv concentration in cells

To estimate the concentration of D3cpv the fluorescence intensity of cells was compared with a pipette filled with eGFP of defined concentration (40 μM, MB-0752, Vector Laboratories). To introduce the pipette a 200 μm opening was carefully drilled into the glass at the very edge of the window. Care was taken not to heat the glass as described for extracellular recording. In animals used for concentration calibration, no functional imaging was done to avoid bleaching or photodamage. As for all experiments, only clear window preparations were used which did not show any blurring effects caused by infections, thickening of dura mater, or re-growth of bone. 30 MΩ pipettes (impedance measured in 3 M KCl) were used. Positive pressure was applied at a level that prevented net flux through the pipette, thus avoiding dilution of the pipette contents or indicator leakage before or after entering the brain. Excitation wavelength was set to 940 nm to principally excite D3cpv's Venus and eGFP but not CFP (two-photon cross sections of 122 GM, 174 GM, and 12 GM respectively, http://www.drbio.cornell.edu/cross_sections.html). Fluorescence intensity of cells and dye in the electrode were compared in the same focal plane as imaging depth influences intensity. Fluorescence intensity was converted to relative XFP concentration by dividing by the photon cross section and the quantum yield of Venus (0.57) (Nagai et al., 2002) or eGFP (0.60) (Patterson et al., 1997).

### Antibody labeling of GABAergic and glycinergic neurons

Putative candelabrum cells were tested for co-localization of GABAergic and glycinergic immunoreactivity. Fixed brain slices 30 μm thick were exposed to GABA antibody (guinea pig, polyclonal, Abcam) and glycine antibody (rabbit, polyclonal, Abcam) and then stained with secondary antibodies labeled with Alexa 488 and Alexa 568 (goat anti-guinea pig IgG, donkey anti-rabbit IgG, both Invitrogen). Images were taken with two-photon microscopy at an excitation wavelength of 970 nm with bandpass filters (510/70 nm for GABA identification and 610/75 nm for glycine identification; Chroma).

### Statistics

All results are given as mean ± SD.

### Conflict of interest statement

The authors declare that the research was conducted in the absence of any commercial or financial relationships that could be construed as a potential conflict of interest.
